# Revealing the Roles of Keratin 8/18-Associated Signaling Proteins Involved in the Development of Hepatocellular Carcinoma

**DOI:** 10.3390/ijms22126401

**Published:** 2021-06-15

**Authors:** Younglan Lim, Nam-On Ku

**Affiliations:** 1 Interdisciplinary Program of Integrated OMICS for Biomedical Sciences, Yonsei University, Seoul 03722, Korea; kr_ran@hanmail.net; 2 Department of Bio-Convergence ISED, Underwood International College, Yonsei University, Seoul 03722, Korea

**Keywords:** keratin, hepatocellular carcinoma, signaling pathway, hepatitis

## Abstract

Although hepatocellular carcinoma (HCC) is developed with various etiologies, protection of hepatocytes seems basically essential to prevent the incidence of HCC. Keratin 8 and keratin 18 (K8/K18) are cytoskeletal intermediate filament proteins that are expressed in hepatocytes. They maintain the cell shape and protect cells under stress conditions. Their protective roles in liver damage have been described in studies of mouse models, and K8/K18 mutation frequency in liver patients. Interestingly, K8/K18 bind to signaling proteins such as transcription factors and protein kinases involved in HCC development. Since K8/K18 are abundant cytoskeletal proteins, K8/K18 binding with the signaling factors can alter the availability of the factors. Herein, we discuss the potential roles of K8/K18 in HCC development.

## 1. Introduction

Globally, liver cancer was the third leading cause of cancer-related death in 2020, and about 90% of liver cancers are hepatocellular carcinoma (HCC) [1,2]. Liver cancer incidences are 905,677 cases and liver cancer-related deaths are 830,180 cases in 2020 in the world, which is 4.7% out of total cancer incidences and 8.3% out of total cancer-related deaths [1]. Liver cancers were more commonly occurred in Asian countries, especially in Eastern and South-Eastern Asia, accounting for about 73% in the last year [1]. Moreover, the incidence of liver cancer is more than twice that in men than in women [1], and particularly, the incidence in the men aged over 50 years old is increasing [3]. The etiology of HCC is relatively well-studied and summarized [2,4]. Hepatitis B virus (HBV) and hepatitis C virus (HCV) infection increases the risk of HCC incidence, and chronic liver diseases, such as cirrhosis and steatohepatitis, also contribute to HCC development [5]. Other than liver diseases, habits such as alcohol abuse and smoking lead to the development of HCC by aggravating liver diseases and/or increasing genetic mutations [5].

Keratins (Ks) are the intermediate filaments that are expressed in the epithelial cells. More than 50 subtypes of keratin are divided into type I (K9-K40) and type II (K1-K8, K71-K86) keratins, and type I and type II keratins form heterodimers with different combinations in a cell type- or tissue-specific manner [6,7]. Specifically, keratin 8 and keratin 18 are the major intermediate filament proteins expressed in hepatocytes [6]. These keratins support cell structure and prevent mechanical stresses by spreading throughout the cytosol [8,9]. K8/K18-related protection has been demonstrated by their mutant effects under in vivo and in vitro systems. Knock-out- or mutation-derived defects of K8/K18 aggravates liver damage and predisposes the liver to certain diseases in transgenic mice [10]. Since chronic liver damage elevates HCC incidence, K8/K18 defects might be one of the factors that can increase HCC incidence. Furthermore, abundant cytoplasmic K8/K18 regulates signaling pathways by interacting with signaling factors and altering their availability and location [11,12]. This K8/K18-mediated regulation can change cell cycle initiation and cell proliferation/migration, which subsequently causes HCC development. In this review, we summarize the general risk factors of HCC development and discuss the potential roles of K8/K18 in altering the risk factors that contribute to HCC incidence.

## 2. Etiology

### 2.1. Liver Diseases

Most HCC occurs in the environment of chronic liver diseases, meaning that HCC is frequently found in patients with cirrhosis and damaged livers due to a hepatitis viral infection or alcohol abuse [2,4,13]. HBV and HCV infections are responsible for half of liver cancer-related deaths [5], and the infections induce chronic inflammation in the liver and increase the risk of HCC [14,15]. The high incidence rate of liver cancers in Asian countries seems to be related to prevalence of HBV and/or HCV infections [16,17]. A study of 1509 patients with HCC demonstrated that vaccination against HBV could decrease HCC incidence [18]. Additionally, HBV infection causes genetic mutations by viral DNA insertion and leads to driver gene mutations [19]. Hence, inflammatory damage and driver gene alteration increase HCC incidence synergistically.

Unhealthy drinking behavior also causes liver damage, and repetitive liver damage induces cirrhosis and dysfunction in the liver [20]. The contribution of alcohol to HCC accounts for 25–30%, and alcoholic cirrhosis is a risk factor for HCC development [5,13]. Patients with alcoholic cirrhosis have been diagnosed as having HCC with an annual incidence of 2.6%, and the 5-year cumulative incidence rate is 13.2% [21]. Notably, excessive drinking markedly increases the risk of HCC in HBV carriers [22]. Taken together, viral infection and excessive alcohol consumption aggravate liver diseases, such as cirrhosis and hepatitis, and cause genetic instability, which leads to the development of HCC.

### 2.2. The Mutations and the Molecular Aspects of HCC Driver Genes 

It has been revealed that alcohol abuse, smoking, and HBV infection are related to gene mutations in HCC [23]. HCC-related cancer driver gene mutations have been analyzed by whole-exome sequencing, and some mutations in the genes cause the alteration of several signaling pathways in HCC [23,24] (Table 1). About 80% of HCC show telomerase reverse transcriptase (TERT)-related gene mutations [23,24]. Telomeres are located at the end of all chromosomes to reduce DNA loss during cell replication. Short telomeres activate cellular response to DNA damage, resulting in cell cycle arrest or apoptosis [25]. Telomerase is a complex that elongates telomere, and consists of TERT, the telomerase RNA component (TERC), and dyskerin [26]. TERT, a key component of telomerase, is expressed in embryonic cells and some stem cells in adults, but not in most cells in adults including hepatocytes [26,27]. Since the shortening of telomeres restricts cell proliferation, telomerase reactivation is required for tumorigenesis [26]. Because of the mutations in the *TERT* promoter and the amplification of *TERT* genes in HCC, telomerase is reactivated and contributes to HCC development.

Mutations of cell cycle regulators are also found in HCC [23,24]. In the case of the p53 transcription factor, the p53 is inactivated in HCC and the regulation of p53-target genes associated with the cell cycle are altered [23,24]. The p53 pathway is stimulated in response to several stresses, such oncogene activation, hypoxia, and genotoxic stress [28]. After its activation, p53 induces apoptosis by activating the transcription of pro-apoptotic proteins, such as BAX, NOXA, and PUMA [29]. Increased apoptosis may inhibit tumorigenesis. In addition to its regulation in cell death, activated p53 can induce cell cycle arrest in response to DNA damage and oncogene activation [30,31,32]. By cell cycle arrest, cells may take time to repair damage and maintain genome stability.

Similarly, mutations in *RB1* (gene of retinoblastoma 1) or *CDKN2A* (gene of cyclin-dependent kinase inhibitor 2A) are frequently found in HCC lesions, and these mutations disturb the regulation of the retinoblastoma (Rb) pathway [23,24,33]. By interacting with E2F, Rb inhibits the transcription of E2F target genes that assist in cell proliferation and the cell cycle. When cyclin-dependent kinases (CDKs) phosphorylate Rb, the phosphorylated Rb loses its regulatory ability, since phosphorylation inhibits the interaction between Rb and E2F [34]. Through this regulation, the Rb pathway induces cell cycle arrest in response to DNA damage [35], and the Rb downregulates cell proliferation [34]. Consequently, the inactivation of Rb causes deregulated DNA replication and cancer progression [36]. For this reason, Rb and CDK inhibitors are known as tumor suppressors. However, the mutations in *RB1* and *CDKN2A* result in the loss of their ability in HCC. As such, cancer development is promoted. 

The inactivating mutations of chromatin remodeling factors, such as *ARID1A/B* and *ARID2*, are found in HCC patients [23,24]. Chromatin remodeling factors assist DNA repair by reorganizing the chromatin structure and, accordingly, the factors that are critical for DNA damage response [37]. A defective DNA damage response may lead to genome instability and tumorigenesis [38,39]; thus, inactivated chromatin remodeling factors may be involved in tumorigenesis. Some studies present more direct evidence indicating that defects in chromatin remodeling factors result in tumor development. A study demonstrated that the chromatin remodeling factor ARID1B suppressed the Wnt-β-catenin pathway (which is described below) [40], and another study described that ARID2 downregulated the epithelial–mesenchymal transition (EMT) of HCC cells [41]. Hence, the deregulation of cell cycle regulators and chromatin remodeling factors may lead to malignant cell proliferation and genome instability, which are both critical for HCC development.

The signaling pathways involved in cell proliferation and/or cell migration are activated by mutations in the Wnt-β–catenin pathway and the Ras–PI3K pathway in HCC patients [23,24]. The Wnt-β–catenin pathway regulates liver development during embryogenesis and liver homeostasis in adults [42]. When Wnt signaling is inactive, β-catenin resides near membrane junctions and some β-catenin can be found in cytoplasm. The β-catenin in the cytoplasm is phosphorylated by the destruction complex, which consists of Axin, adenomatous polyposis coli (APC), GSK3β, and CK1α, followed by the degradation of β-catenin [43]. When the Wnt protein, which comes from the outside of cells, binds to its receptors on the membrane, β-catenin is released from the destruction complex. The free β-catenin translocates into the nucleus and acts as a regulator of activators and transcription factors of genes related to cell proliferation (including MYC and growth factors), EMT, and inflammation (including IL-6 and IL-8) [42]. Hence, β-catenin is the key regulator of the Wnt signaling pathway. In early stages of HCC, β-catenin resides near the plasma membrane and upregulates the activity of growth factor receptors [44], while in late stages of HCC, nuclear accumulation of β-catenin is frequently observed [45]. It shows the complexity of the roles of β-catenin in HCC development. Furthermore, the genetic mutations of β-catenin proteins and/or the components of the destruction complex, such as Axin and APC, are commonly found in HCC patients [23,24]. These mutations inhibit the negative regulators (Axin and APC) and enhance β-catenin activation so that the Wnt-β-catenin pathway is excessively activated. Since the Wnt-β-catenin pathway is involved in cell proliferation and EMT, its aberrant activation leads to HCC development [46,47].

Aberrant activation is also observed in the Ras-PI3K pathway through the gene mutations in *NRAS*, *KRAS*, *PIK3CA*, and *PTEN* [23,24,48]. Ras is a well-studied oncogenic protein since its activates several pathways, including the PI3K-Akt pathway, that regulate cell proliferation and cell growth [49]. When Ras is activated, PI3K phosphorylates PIP_2_, PIP_3_, and PIP_3_ activate downstream proteins, such as PDK and Akt, that eventually induce cell survival and proliferation and downregulate apoptosis [49]. This signaling pathway can be reversed by PTEN, the phosphatase that convert PIP_3_ to PIP_2_. Thus, the constitutive activation of Ras family proteins induced by *NRas and KRAS* mutations may participate in tumor development [23,24,50]. In the same way, abnormal activation of the PI3K-Akt pathway can be observed by the inhibiting mutations of *PTEN* and the activating mutations of *PIK3CA* [24], and IRS-4, which is the upstream activator of Akt pathway, translocates into nucleus in tumor tissues, while IRS-4 is located in the cytosol and membrane in healthy tissues [51]. Higher expression of IRS-4 is correlated with increased proliferation, which might lead to tumorigenesis in HCC [51]. Unregulated Wnt and Ras-PI3K pathways increase the risk of tumor development and malignancy. Taken together, the mutations of genes involved in signaling pathways lead to perturbed cell cycle regulation and cause the increased activation of cell migration, proliferation, and telomerase. These alterations of cellular events may increase HCC incidence.

## 3. HCC Management

HCC treatment can be divided into surgical treatment, radiotherapy, and chemotherapy. The therapies are adjusted according to tumor stages following the Barcelona clinical liver cancer (BCLC) score [16,52]. Usually, patients with early-stage tumors are the candidates for surgical treatments, such as resection, transplantation, and local ablation [16,52]. Hepatic resection can be effective when the patients do not have cirrhosis [2]. Patients with intermediate-stage tumors are candidates for trans-arterial chemoembolization (TACE) [2,16,52,53]. TACE is the method by that the specific hepatic artery is blocked and thus nutrients and oxygen cannot be provided to tumor tissues [54]. Patients with more advanced stage tumors become candidates for chemotherapy or combination therapy [55]. There are several FDA-approved drugs for HCC. For first-line therapy, Sorafenib, Lenvatinib, and recently Bevacizumab + Atezolizmab are the standard treatments. Regorafenib, Cabozantinib, Ramucirumab, and Nivolumab + Ipilimumab are used for second-line therapy. Sorafenib is a multi-kinase inhibitor that inhibits the activity of Raf, VEGFRs, and PDGFRs [2,56]. By inhibiting them, cell proliferation and angiogenesis are inhibited. Sorafenib has been the standard treatment for HCC patients [52]. Similarly, Lenvatinib, Regorafenib, Cabozantinib, and Ramucirumab inhibit the subtypes of VEGFRs [48,57,58]. By down-regulating angiogenesis, the drugs can inhibit tumorigenesis and/or down-size the tumor tissues. Recent studies target for immune checkpoints to eliminate cancer cells by immune cells and examine the chemotherapy using two different types of drugs such as Bevacizumab + Atezolizmab and Nivolumab + Ipilimumab [2,59]. Pairing immune checkpoint inhibitors with anti-angiogenic drugs seems promising to HCC treatment, and pairing of immune checkpoint inhibitors and other immune check point inhibitors is also used for several clinical trials [2,59] (Table 2). The clinical trials have been reported at the database ClinicalTrials.gov. By using the combination of surgical treatment, radiotherapy, and chemotherapy, HCC can be managed, but still it needs more investigations of treatment for patients with advanced stage of HCC.

## 4. Keratin 8/18 and Liver Diseases

### 4.1. K8/K18 Mutations Associated with Liver Diseases

Keratins are intermediate filament proteins expressed in the epithelial cells. As cytoskeletal proteins, keratins maintain cell shape, support the structure from external mechanical stress, and regulate signaling pathways by interacting with signaling proteins or restricting the availability of signaling proteins [7,60]. They are expressed as a pair of type I and type II keratins in a tissue-specific manner, and K8 and K18 are the only pair of keratins found in the liver [6,7]. The protective roles of K8/K18 in the liver have been determined by knock-out or transgenic mouse systems (Table 3). K8 knock-out mice with a C57/B1 genetic background are 94% embryonically lethal, while mice with an FVB/n genetic background survive and have mild hepatitis under basal conditions [61,62]. K8-null mice are predisposed to liver damages that are induced by Fas injection or a specific diet [63,64,65]. Likewise, K18-null mice experience liver hemorrhages under basal conditions, and old K18-null mice (17–20 months) spontaneously develop steatohepatitis and liver cancers [66,67].

**Figure 1:**
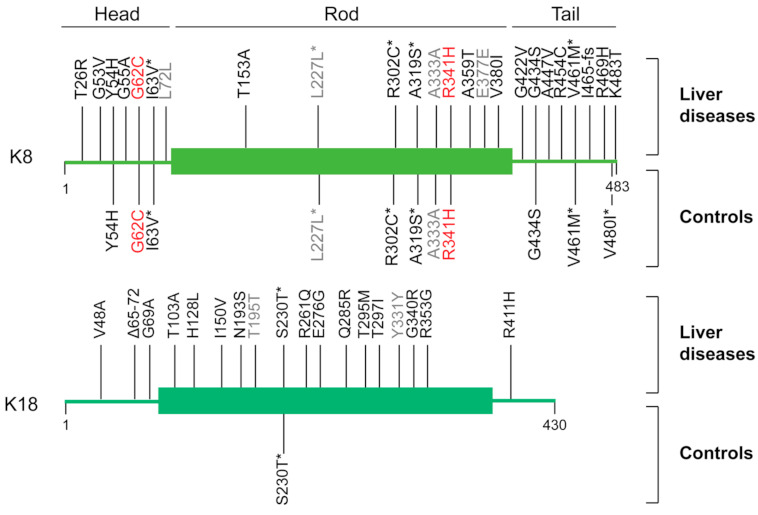
Distributions of K8/K18 variants found in patients with liver diseases and controls: The protein subdomains and amino acid positions of K8 and K18 are indicated. The K8/K18 variants are illustrated on a diagram of K8/K18. The variants in red are the mutations studied with transgenic mouse models, while the variants in grey are silent mutations. The mutations with an asterisk (*) indicate polymorphisms, since the mutations have been found in the liver disease groups and the controls with similar mutation frequency. The K8/K18 mutations were analyzed in the studies of Strnad P et al., Li R et al., Zhong B et al., Ye J et al., and Ku N.O. et al. [83,84,85,86,87,88,89,90].

The skin-specific keratin 14 mutation K14 R125C was identified in patients with skin diseases [82]. A K18 R90C mutation is equivalent to a K14 R125C mutation. Neither K14 R125C nor K18 R90C can form a normal filament network in the skin and the liver, respectively [78,82]. Studies of K18 R90C transgenic mice show that K18 R90C mutation predisposes transgenic mice to liver diseases [78,79,80] (Table 3). Since these studies, K8/K18 mutations have been analyzed in patients with liver diseases, such as hepatitis, cirrhosis, and liver cancers [83,84,85,86,87,88,89,90] (Table 4, Figure 1). Most studies demonstrate that K8/K18 mutation frequency is correlated with disease incidence, as shown in Table 4, and various mutations of K8 and K18 have been identified in patients with liver diseases (Figure 1). A previous study shows that the missense mutation frequency of the K8 coding region is higher in patients with primary liver cancer (PLC) compared to controls, although it is not statistically relevant (PLC vs. controls; 2.3% vs. 0.58%, *p* = 0.179) [83]. Notably, the intron variant frequency of K8 is correlated with PLC compared to controls (PLC vs. controls; 2.3% vs. 0%, *p* = 0.038), although the physiological functions of the intron variants still need to be determined [83]. The analysis of keratin variants in liver diseases demonstrates the interconnection between the frequency of keratin variants and liver diseases.

The correlation between K8/K18 variants and liver damage has been described in studies of transgenic mice expressing the keratin mutants identified in patients with liver diseases. The studies described K8 R341H and G62C, which are frequently found mutations in the livers of patients [10]. K8 R341H, the most frequently found mutation, may destabilize the keratin structure, according to predicted structural consequences [88]. Its significance in liver protection has been described with K8 R341H transgenic mice, which are susceptible to acute acetaminophen (APAP) treatment [73]. K8 G62C introduces cysteine residues instead of glycine residues. It is distinctive, considering that wildtype K8 does not contain a cysteine residue. Since the K8 G62C mutation resides near K8 S74, a major phosphorylation site on K8, the K8 G62C mutation inhibits K8 S74 phosphorylation [91]. The significance of K8 G62C mutation has also been demonstrated in the transgenic mouse system. K8 G62C or K8 S74A transgenic mice develop severe liver damage under stressed conditions [91]. In summary, K8/K18 mutations have been identified at a higher frequency in liver patients compared to controls, and natural keratin variants, such as K8 G62C and K8 R341H, exacerbate liver damage in transgenic mice. Since repetitive liver damage may lead to HCC development, K8/K18 variants seem to be related to HCC risk.

### 4.2. K8/K18-Related Inclusion Body and K18 Apoptotic Fragment as Liver Disease Biomarkers

Abnormal protein aggregates are easily found inside the cells of some diseases, such as Alzheimer’s disease, Parkinson disease, and liver diseases [92,93,94]. Protein aggregates, also called inclusion bodies, are made up of misfolded structural proteins with chaperones and the ubiquitin-binding protein p62 [94]. The inclusion bodies are divided into different types depending on the types of structural protein in the aggregates. K8/K18 in the liver are involved in the formation of the inclusion bodies named Mallory-Denk bodies (MDBs). MDBs are easily found in the cytoplasm of hepatocytes with various liver diseases, such as alcoholic and non-alcoholic steatohepatitis and primary biliary cirrhosis [69,94,95]. Notably, about 50% of HCCs contain MDBs and/or intracellular hyaline bodies (IHBs), which are similar to MDBs except that keratins are not contained [96].

The proteins in inclusion bodies are often hyper-phosphorylated, and keratin phosphorylation may be important for the formation of MBDs [94,97]. After mice were fed the diet that induces MDB formation, phosphorylated keratins were accumulated in insoluble fractions [98]. Some studies have revealed that keratin phosphorylation induces MDB formation. Microcystin-LR treatment, which increases K8/K18 phosphorylation, induces MDB formation [99], and the treatment of the phosphatase inhibitor okadaic acid increases inclusion bodies in the mouse liver [100]. Conversely, a study has revealed that K8 G62C or K8 S74A transgenic mice that show inhibited phosphorylation on K8 S74 display a decreased formation of MDBs [101]; these transgenic mice are highly susceptible to liver stresses [91]. In addition, by inhibiting p38, which is a kinase that phosphorylates K8 S74 residues, MDB formation can be decreased [102]. Thus, the phosphorylation of keratins is associated with MDB formation. Along with phosphorylation, the ratio of K8/K18 is critical for MDB formation. Previous studies have revealed that the increased ratio of K8/K18 is related to the production of MDBs [69,94,103]. MDB formation increases in K8 overexpression or K18 knock-out mice, while the formation does not change in mice overexpressing both K8 and K18 [69,103,104,105]. Even K18 overexpression inhibits MDB formation and eventually protects the liver from a toxic diet in mice [69]. It is likely due to cross-ß sheet structure of keratin 8 during MDB formation [105].

In terms of a relation between MDB formation and signaling pathways, several studies have demonstrated that the BRCA1-mediated signaling pathway and the G1-S cell cycle checkpoint pathway are upregulated in alcoholic hepatitis containing MDBs compared with healthy livers [106]. Furthermore, the inflammatory response is increased by the upregulation of the IL-8-related pathway in the MDB-featuring livers of DDC fed-mice and human alcoholic hepatitis [107,108], as well as in the NF-kB signaling pathway in primary hepatocytes with MDB-inducing agents [107,108]. MDB formation in primary hepatocytes with MDB-inducing agents specifically induces inflammation by the enhanced NF-kB signaling pathway via IkB sequestration in hepatic protein aggregates of MDB [108]. Notably, the accumulation of p62, which is a component of inclusion bodies, increases the activity of transcription factor Nrf2, thereby causing liver damage [109]. The aberrant activation of Nrf2 concurs with HCC-related driver gene mutations. The gene of Nrf2, *NFE2L2*, is mutated in a manner that enhances its function, and these mutations are partly responsible for HCC development [24,33] (Table 1). Therefore, MDB formation in liver lesions is a biomarker for severe liver diseases, and increased MDB formation may be associated with aggravated hepatitis that could possibly enhance HCC development.

A K18 apoptotic fragment can be used for the diagnosis of liver disease. Aberrant cell death contributes to the progression and severity of liver diseases [110]. During apoptosis, K18 is cleaved at K18 D238 and D397 residues by activated caspases [111,112], and this K18 cleavage produces fragments of K18 that are released into serum or plasma [6]. The K18 fragments are then detected by M30 ELISA, and total K18 is detected by M65 ELISA. Then, the higher amount of M30 or M65 and the higher ratio of M30 to M65 are each correlated with the disease score and apoptosis in various liver diseases [6,113,114,115,116,117]. In terms of HCC, a recent study demonstrated that the K8/K18 ratio can be used as a biomarker for HCC and its early recurrence [118]. Since the levels of K8, K18, and K18 fragments can be measured with serum or blood, K8/K18 can be used as effective diagnostic tools for liver damage.

## 5. Keratin 8/18-Related Signaling Pathways

K8/K18 protect hepatocytes through the structural support and regulation of signaling pathways [7,85,119]. The protection that comes from structural support has been demonstrated by studies with K18 R90C mice [78] (Table 3). Here, we summarize K8/K18-associated signaling proteins that may be involved in HCC development (Table 5).

Studies of cancer driver gene mutations show that cell cycle control seems to be a key regulator of HCC development. Previous studies of K8/K18 knock-out or K18 mutations in transgenic mouse model systems describe the effects of keratins in cell cycle regulation [62,76]. In K8- or K18-null mice, which lack both K8 and K18 in the hepatocytes, and K18 R90C mice, which have disrupted keratin filament, multinuclear giant cells were found in abnormal liver lesions, and a high proportion of the cells were in the S-G2 phases of the cell cycle [62]. Protein 14-3-3 is known to control cell cycle regulatory proteins by sequestration [123,124], and the cellular localization of 14-3-3 proteins can be changed by their interaction with K18 [123,124]. Since the distribution of 14-3-3 proteins has been altered in K8-null livers compared to K8 WT livers, it is likely that the absence of normal keratin filament in liver disturbs the cell cycle regulation [62]. Similarly, K18 S34A transgenic mice have more mitotic features in the liver compared to WT mice, which indicates more mitotic arrest in K18 S34A livers [76]. Since 14-3-3 protein binds to K18 S34 residues in a phosphorylation-dependent manner [123], the altered cell cycle in K18 S34A transgenic mice may be caused by the release of 14-3-3 from K8/K18. Taken together, K8/K18 may be involved in the regulation of cancer development by cell cycle regulation via 14-3-3 sequestration in the cytosol (Table 5).

K8/K18 might be involved in the regulation of HCC development by altering cell proliferation and migration signaling pathways through K8/K18-associated signaling proteins such as Akt and Raf, as well as stress-activated protein kinases (SAPKs). The PI3K/Akt pathway is one of the well-known signaling pathways in tumorigenesis, since Akt has a critical role in cell proliferation, migration, and anti-apoptosis. A study showed that K8 interacts with Akt under basal conditions and Akt activity was downregulated in K18 glycosylation-deficient transgenic mice (K18 S30/31/49A) [75] (Table 5). The study showed that blocked K18 glycosylation induced Akt hyper-glycosylation, followed by inhibited Akt T308 phosphorylation [75]. Although the detailed molecular mechanisms remain to be determined, K8/K18 may, at least in part, regulate the Akt pathway.

The SAPK pathways can be altered by the disruption of the phosphorylation of K8 [60] (Table 5). K8 is known as a substrate of SAPKs, and K8 S74 and S432 are the residues that are phosphorylated by SAPKs [76,125]. Due to the abundance of K8 in cells, inhibited phosphorylation on K8 S74 in K8 G62C or blocked phosphorylation in K8 S74A results in the increased availability of SAPKs to other substrates, such as CREB and c-JUN, and leads to an increased phosphorylation level of the SAPK substrates [60]. Since SAPKs regulate cellular events in response to stresses, the deregulation of SAPKs leads to the severe liver damage shown in K8 G62C and S74A transgenic mice under stress conditions [60]. Hence, it seems that the disturbed phosphorylation on K8/K18 aggravates liver damage that may contribute to HCC development. Furthermore, the binding site of p38, known as one of the SAPKs, on K8 has been revealed [120]. Positively charged residues on K8 (K8 R148/149, L159/161) are responsible for the p38 interaction; thus, K8 R148/149E, L159/161A mutation blocks the interaction, and K18 I150V, a natural mutation found in patients with liver diseases, inhibits the interaction due to its proximity to the binding site of p38 in the form of a K8/K18 heterodimer [120]. The dissociation of p38 with K8/K18 leads to nuclear localization of phosphorylated p38 and higher transcription of p38-dependent target genes under cultured cell transfection systems [120]. Taken together, the K8/K18-p38 association regulates the localization of SAPK and may thereby alter the phosphorylation and transcription levels of downstream factors of SAPKs.

The Ras-Raf pathway and protein kinase C (PKC) pathway are possibly affected by K8/K18 (Table 5). A study reveals that K8/K18 interacts with Raf, and that the interaction is dissociated under hyperphosphorylation or stressed conditions [121]. It can be assumed that the physical interaction of Raf with K8/K18 might perturb the interaction of Ras-Raf, which is required to induce the activation of the Ras-Raf pathway. PKC is involved in cell migration [126], and PKC activity can be increased by its interaction with the anchoring protein RACK1 [127,128]. RACK1 is sequestrated to the cytoskeleton, including intermediate filament keratins, via plectin; thus, the absence of this sequestration leads to the translocation of RACK1 and to increased degradation of PKC [129,130]. Indeed, K8/K18, as cytoskeletal proteins, can affect the PKC pathway by regulating the localization of plectin and RACK1, and thereby cell migration [122]. It seems likely that K8/K18 regulate cell proliferation/migration by an altered Raf pathway and/or a PKC-mediated pathway.

In terms of keratin-associated transcription factors, p53 interacts with K8/K18 under basal conditions, and p53 is dissociated from K8/K18 under hyperphosphorylated conditions [70] (Table 5). Protein p53 is known as a tumor suppressor since p53 is involved in apoptosis and cell cycle arrest, and genetic mutations of the *TP53* gene, which encodes p53, are found in HCC patients [24,33,131]. It is well-known that p53 mutation is highly related to HCC development. Since K8/K18 are highly abundant proteins, the interaction between p53 and K8/K18 might alter p53 availability and, by extension, the progress of HCC development. The NF-kB transcription factor is also a binding protein of K8/K18 under basal conditions while being dissociated under stressed conditions [70] (Table 5). In K8-null mice under stressed conditions, NF-kB is downregulated and its nuclear translocation is inhibited [70]. Since NF-kB is required for cell survival [132], the downregulation of NF-kB may enhance the severity of liver damage in K8-null mice under stressed conditions. It is likely that intact K8/K18 are essential for proper NF-kB activation. In summary, K8/K18 interact with signaling proteins and thereby regulate their availability, which may lead to the regulation of the cell cycle, as well as to proliferation, migration, or apoptosis. These altered cellular events may affect the incidence of liver damages and HCC.

## 6. Conclusions

HCC occurs with complex etiologies, such as genetic alterations and chronic liver damage [2,4]. Chronic liver diseases can be induced by virus infection (HBV and HCV) and environmental factors (alcohol intake and smoking), and chronic liver diseases and genetic mutations in the lesions lead to an increase in HCC development [2,4]. Although the HCC incidence induced by viral infection can be reduced by vaccination, the incidence of liver cancers is growing annually [5,18]. It needs additional biomarkers and novel targets that provide potential prognostic information for HCC. Interestingly, a recent study suggests the K8/K18 ratio as a new biomarker for liver cancers [118], although the detailed molecular mechanism remains to be determined. On the other hand, previous studies about K8/K18 binding proteins show that K8/K18 interact with various signaling molecules in different signaling pathways, including cell proliferation, migration, and apoptosis (Table 5). Since K8/K18 are abundant cytoskeletal proteins, K8/K18 binding with signaling factors can alter the availability and location of the factors that may be involved in HCC development.

## Figures and Tables

**Table 1:** Altered cancer driver genes in hepatocellular carcinoma.

Pathway	Gene	Alteration	Ref.
Telomere maintenance	*TERT* promoter	Gain of function	[23,24]
Cell cycle	*TP53*	Loss of function	
	*RB1*	Loss of function	
	*CDKN2A*	Loss of function	
Chromatin remodeling	*ARID2*	Loss of function	
	*ARID1A/B*	Loss of function	
Wnt pathway	*CTNNB1*	Gain of function	
	*AXIN1*	Loss of function	
Ras-PI3K pathway	*PIK3CA*	Gain of function	
	*RPS6KA3*	Unclassified	
	*PTEN*	Loss of function	
	*KRAS*	Gain of function	
	*NRAS*	Gain of function	
Oxidative stress	*KEAP1*	Gain of function	
	*NFE2L2*	Gain of function	

Data are summarized from Schulze, K. et al. [23] and Ally, A. et al. [24]. Both studies show the identified mutations of HCC driver genes by whole-exome sequencing analysis in 243 liver tumors [23] and 363 HCC cases [24].

**Table 2:** Combination of immune checkpoint inhibitor therapies in clinical trials.

Drug	Targets	HCC Stage	Phase	Comparison	Primary Outcome	NCT Number ^‡^
Atezolizumab + Lenvatinib	PD-L1 + VEGFRs, FGFRs, PDGFRβ, RET, and KIT	Advanced or metastatic	ΙΙΙ	Sorafenib	OS	NCT04770896
Toripalimab + Lenvatinib	PD-1 + VEGFRs, FGFRs, PDGFRβ, RET, and KIT	BCLC B or C stage	ΙΙ	– ^†^	ORR	NCT04368078
SHR-1210 + Apatinib	PD-1 + VGFR-2	Advanced	ΙΙΙ	Sorafenib	OS/PFS	NCT03764293
Durvalumab + Bevacizumab	PD-L1 + VEGF-A	High risk of recurrence	ΙΙΙ	Placebo	RFS	NCT03847428
Nivolumab + Ipilimumab	PD-1 + CTLA-4	Advanced	ΙΙΙ	Sarafenib or Lenvatinib	OS	NCT04039607
Pembrolizumab + Bavituximab	PD-1 + PS	Advanced or metastatic	ΙΙ	– ^†^	ORR	NCT03519997
Durvalumab + Tremelimumab	PD-1 + CTLA-4	Advanced	ΙΙΙ	Sorafenib	OS	NCT03298451

^†^ Non-randomized or single-arm trials; ^‡^ National clinical trial identifier, which is a unique code for each trial study. Data were acquired in June 2021 from the database ClinicalTrials.gov. The combination therapy of immune checkpoint inhibitor + another immune checkpoint inhibitor or immune checkpoint inhibitors + anti-angiogenic drugs was selected from recently reported clinical trials. BCLC, Barcelona-clinic liver cancer; PD-1, programmed cell death; VEGFR, vascular endothelial growth factor receptor; VEGF, vascular endothelial growht factor; PD-L1, programmed cell death ligand 1; FGFR, fibroblast growth factor receptor; CTLA-4, cytotoxic T lymphocyte-associated antigen-4; PS, phosphatidyl serine; OS, overall survival; PFS, progression free survival; ORR, objective response rate.

**Table 3:** Liver phenotypes of K8/K18-related mouse models.

Gene	Mouse Genotype	Keratin Filament	Liver				Ref.
			Basal Phenotype	Fragility (Basal Conditions)	Phenotype (Stressed Conditions)	Induced Stresses	
K8	K8-/- (C57B1/6)	Absent	Embryonic lethality Liver hemorrhage	-	-	-	[68]
	K8-/- (FVB/n)	Absent	Mild hepatitis	↑	↑, Decreased MDBs	Pentobarbital, MLR, high fat diet, Fas	[61,63,65,69,70,71]
	K8 over-expression	Normal	MDBs	Normal	Increased MDBs	DDC, high fat diet	[69,72]
	K8 G62C	Normal	Normal	Normal	↑	Fas, MLR, APAP	[60,73]
	K8 S74A	Normal	Normal	Normal	↑	Fas	[60]
	K8 R341H	Normal	Normal	Normal	↑	APAP	[73]
K18	K18-/-	Absent	Mild hepatitis MDBs Steatohepatitis (Old mice)	↑	↑	Fas	[66,67,74]
	K18 over-expression	Normal	Normal	Normal	Decreased MDBs	DDC	[69]
	K18 S30/31/49A	Normal	Normal	Normal	↑	STZ, Fas + PUGNAc	[75]
	K18 S34A	Normal	Normal	Normal	Mitoticfeatures	PH	[76,77]
	K18 S53A	Normal	Normal	Normal	↑	MLR	[10]
	K18 R90C	Disrupted	Mild hepatitis	↑	↑	Fas, CCl_4_, TAA	[78,79,80]
	K18 D238/397E (mouse K18expressed FVB/n)	Normal	Normal	Normal	↑	Fas	[81]
		Normal	Normal	Normal	-	Fas	[74]
	K18 D238/397E (mouse K18 knocked out FVB/n)	Normal	Normal	Normal	↓	Fas, MLR	[74]

APAP, acetaminophen; DDC, 3,5-diethoxycarbonyl-1,4-dihydrocollidine; MDB, Mallory-Denk body; MLR, microcystin-LR; PH, partial hepatectomy; PUGNAc, 1,5-hydroxymolactone; STZ, streptozotocin; TAA, thioacetamide. ↑upregulated; ↓downregulated.

**Table 4:** Comparison of keratin variant frequency in patients with liver diseases and controls.

Screened Gene	Ethnicity	No. of Variant Carriers/Total (%)		*p* Value	Ref.
		Liver Disease Cohort	Controls		
AllK8/K18 exons	US	58/467 (12.4%)	13/349 (3.7%)	<0.0001	[87,88]
	Germany	19/329 (5.8%)	-	0.001	[89]
	US	45/344 (13.1%)	9/268 (3.4%) ^a^	0.01 ^a^	[83]
	Italy	17/201 (8.5%)	4/200 (2%)	*p* < 0.004	[85]
	China	10/200 (5%)	1/173 (0.58%)	*p* = 0.015	[84]
	China	21/540 (3.89%)	1/173 (0.58%)	*p* = 0.03	[86]
K8 exon 1 and 6	Germany	12/151 (7.9%)	-	-	[90]

^a^ The mutation identification was performed with white controls and African American controls, but the number of mutant carriers of controls and *p* value were marked with the numbers in white people because only the K8 exon 1, 6, and 8 were analyzed in African American controls. (9.1% vs. 3.4%; white patients vs. white controls).

**Table 5:** K8/K18-associated proteins in signaling pathways.

A Role of Signaling Protein	K8/K18-Associated Signaling Protein	K8/K18 Mutation	Effects of K8/K18 Mutation	Ref.
Cell cycle regulator	14-3-3	K8-/- K18-/- K18 R90C (disrupted filament)	Arrest in S-G2 phage (in vivo) ^ǂ^	[62]
		K18 S34A (blocked pK18 S34, disturbed 14-3-3 binding)	Accumulation of mitotic figures (mitotic arrest) (in vivo) ^ǂ^	[76]
Transcription factor	p53	-	ND ^#^	[70]
	NF-kB	K8-/-	High susceptibility to Fas treatment Inhibited NF-kB translocation to the nucleus	[70]
Kinase	Stress-activated protein kinases (SAPKs, such as ERK, JNK, and p38)	K8 G62C * (natural mutation, inhibited pK8S74) K8 S74A(blocked pK8 S74)	High susceptibility to Fas treatment Higher activation of SAPK substrates (in vivo) ^ǂ^	[60]
		K8 R148/149E, L159/161A (blocked p38 binding) K18 I150V * (natural mutation)	Dissociation of p38 Translocation of phosphorylated p38 to nucleus (in vitro) ^ʌ^	[120]
	Protein kinase B (PKB also known as Akt)	K18 S30/31/49A (K18 glycosylation-deficient mutant)	Akt hyper-glycosylation Inhibition of Akt T308 phosphorylation (in vivo) ^ǂ^	[75]
	Raf	-	ND ^#^	[121]
	Protein kinase C (PKC)	K8-/-	Reduced migration of liver epithelial cells (in vitro) ^ʌ^	[122]

* Natural mutations identified in patients with various liver diseases; ^ǂ^ in knockout or transgenic mouse system; ^ʌ^ in cultured cell system; ^#^ not determined.

## Abbreviations

APAP	acetaminophen
APC	adenomatous polyposis coli
BCLC	Barcelona clinical liver cancer
CDK	cyclin-dependent kinase
EMT	epithelial-mesenchymal transition
G	globular
HBV	hepatitis B virus
HCC	hepatocellular carcinoma
HCV	hepatitis C virus
IHB	intracellular hyaline body
K	keratin
MDB	Mallory-Denk body
PKB	protein kinase B
PKC	protein kinase C
PLC	primary liver cancer
Rb	retinoblastoma
SAPK	stress-activated protein kinase
TACE	trans-arterial chemoembolization
TERT	telomerase reverse transcriptase
TERC	telomerase RNA component
